# Gut microbiome functionality might be associated with exercise tolerance and recurrence of resected early-stage lung cancer patients

**DOI:** 10.1371/journal.pone.0259898

**Published:** 2021-11-18

**Authors:** Andrea Marfil-Sánchez, Bastian Seelbinder, Yueqiong Ni, Janos Varga, Judit Berta, Virag Hollosi, Balazs Dome, Zsolt Megyesfalvi, Edit Dulka, Gabriella Galffy, Glen J. Weiss, Gianni Panagiotou, Zoltan Lohinai

**Affiliations:** 1 Systems Biology and Bioinformatics, Leibniz Institute for Natural Product Research and Infection Biology, Hans Knöll Institute, Jena, Germany; 2 National Koranyi Institute of Pulmonology, Budapest, Hungary; 3 Department of Thoracic Surgery, National Institute of Oncology-Semmelweis University, Budapest, Hungary; 4 Division of Thoracic Surgery, Department of Surgery, Comprehensive Cancer Center, Medical University of Vienna, Vienna, Austria; 5 County Hospital of Torokbalint, Torokbalint, Hungary; 6 MiRanostics Consulting, Oro Valley, Arizona, United States of America; University of Illinois Urbana-Champaign, UNITED STATES

## Abstract

Impaired exercise tolerance and lung function is a marker for increased mortality in lung cancer patients undergoing lung resection surgery. Recent data suggest that the gut-lung axis regulates systemic metabolic and immune functions, and microbiota might alter exercise tolerance. Here, we aimed to evaluate the associations between gut microbiota and outcomes in lung cancer patients who underwent lung resection surgery. We analysed stool samples, from 15 early-stage lung cancer patients, collected before and after surgical resection using shotgun metagenomic and Internal Transcribed Spacer (ITS) sequencing. We analysed microbiome and mycobiome associations with post-surgery lung function and cardiopulmonary exercise testing (CPET) to assess the maximum level of work achieved. There was a significant difference, between pre- and post-surgical resection samples, in microbial community functional profiles and several species from *Alistipes* and *Bacteroides* genus, associated with the production of SCFAs, increased significantly in abundance. Interestingly, an increase in VO_2_ coincides with an increase in certain species and the "GABA shunt" pathway, suggesting that treatment outcome might improve by enriching butyrate-producing species. Here, we revealed associations between specific gut bacteria, fungi, and their metabolic pathways with the recovery of lung function and exercise capacity.

## Introduction

The ethology or progression of lung cancer has been linked to numerous factors, including the gut microflora [1]. The microbiome can control epithelial cell proliferation/ differentiation, nutrition, detoxification, metabolism, and hormonal homeostasis. Gut microbiota has been implicated in tumour development in cancers [2, 3]. It has been shown that several chronic lung diseases, including cancer may be linked to a dysbiotic airway microbiota and frequently occur in conjunction with gastrointestinal disorders [4].

Previous studies showed that exercise training can alter the abundance of butyrate in the gut through an increase in the relative abundance of butyrate-producing species [5, 6]. Butyrate is a short-chain fatty acid (SCFA) that can regulate proliferation of epithelial cells in the gut, improve the integrity of the gut barrier, and alter the immune system and gene expression of the host [6]. A decrease in the abundance of Firmicutes has been repeatedly reported in lung cancer patients [2, 7] and has been previously associated with dysbiosis of gut metabolism, resulting in lower SCFAs concentration [2]. In contrast, a higher ratio of Firmicutes to Bacteroidetes bacteria has been show to correlate with maximal oxygen uptake during exercise (VO_2_) [8]. Barton *et al*. [9] showed that athletes have greater faecal SCFA concentrations and altered abundance of bacterial pathways related to the biosynthesis of amino acids and the metabolism of carbohydrates. Others found that exercise training can alter the gut microbiota, at the taxonomic and functional level, in obese and non-obese humans [10] and reduce the expression of genes associated to metabolism of fructose and amino acids [11]. Moreover, exercise training may affect the gut mucus layer integrity, which is involved in the prevention of microbial adherence to the gut epithelium and acts as a substrate for bacteria of the mucosa-associated microflora. Increased heat shock protein response that prevents tight junction breakdown between epithelial cells [12] and improves resilience of the gut barrier [13, 14] has been observed in trained athletes. Others showed that physical activity decreases the risk for colorectal cancer by 24% [15].

Cardiopulmonary exercise testing (CPET) is a non-invasive, physiological test that provides a comprehensive overview of the pulmonary response to exercise and allows for the evaluation of the body’s metabolic state, functional capacity, and impairment, through the assessment of submaximal and peak exercise responses. The metabolic changes that characterize health status and tumour recurrence have a stronger correlation with exercise tolerance, as measured by CPET, than with resting pulmonary and cardiac function testing, thus resulting in an increase in the use of CPET in patient management. Among many parameters, CPET measures the oxygen uptake (VO_2_) peak that is used in predicting postoperative overall condition and pulmonary complications [16, 17].

In this study, our primary aim was to characterize the taxonomy and functionality of the gut microbiomes’ metabolic interaction in lung cancer patients who underwent lung resection surgery. Our secondary aim was to describe the taxonomy changes in the same setting according to postoperative disease recurrence. We focus on the alterations of microbiome functionality to understand the complex gut-lung axis’ metabolic interactions.

## Materials and methods

### Study population

From 2018 to 2019, we initially screened a cohort of 98 lung cancer patients from the National Koranyi Institute of Pulmonology and County Hospital of Torokbalint. From these, 20 were in early-stage and had their tumours surgically resected. Ultimately, 15 cases were suitable for the CPET testing and underwent pulmonary rehabilitation. A flow chart of study participants and excluded patients is shown in S1 Fig. Baseline and followed-up samples were collected from those patients. Clinicopathological data collected included gender, age, smoking history, chemo- and radiotherapy treatments, type of operation, and overall survival (OS) or disease-free survival. Tumour, node, metastases stage according to the Union for International Cancer Control (8th edition [18]), and age at the time of diagnosis were recorded. The study and all treatments were conducted in accordance with the guidelines of the National Comprehensive Cancer Network and the Helsinki Declaration of the World Medical Association. The study was approved by the national level ethics committee (Hungarian Scientific and Research Ethics Committee of the Medical Research Council (ETTTUKEB- 50302-2/2017/EKU)). Informed consent was obtained for all patients. Stool samples were collected before (prior to surgery within 14 days) and 12 months after lung resection surgery. To assess a patient’s overall condition, we performed a CPET test one year post-surgical resection to coincide with the time of follow-up stool sample collection. Major characteristics of the patient cohort are displayed in S1 Table. Patients received no neo-adjuvant therapy, and selected cases were treated with postoperative systemic chemotherapy with a platinum-based doublet regimen therapy (S1 Table).

### Pulmonary function (PF) and CPET testing

We used spirometry at rest one year after surgery to assess key lung function parameters including {percentage of reference value} (Forced Expiratory Volume (FEV1) {%}), total Lung Capacity (TLC{%}), residual volume (RV{%}).

We performed CPET that begins with mild followed by intense exercise on an upright bicycle. Patients breathing through a mouthpiece. We measured the ventilation and respiratory gas parameters during exercise using oxygen and carbon dioxide gas analysers. Respiratory volumes were computed by integrating the air flow signals over the time of inspiration and expiration. The CPET test lasted until the maximal effort workload achieved for approximately 10 minutes.

We performed an incremental exercise test on an electronically braked cycle ergometer (Ergoline-900, Marquette) that begin with 3 minutes rest and 3 minutes constant pedalling at 20 W, work rate was increased from 5, 10, or 15 W/min in ramp profile. Patient pedalling speed was 60 rpm. Pulmonary ventilation (VE) and gas exchange (VO_2_ and carbon dioxide output (VCO_2_)) were measured breath-by-breath by a mass flow sensor and exercise metabolic measurement system (Vmax 29c, SensorMedics). During the test, we monitored heart rate (HR) using a 12-lead ECG (Cardiosoft, SensorMedics) and oxygen saturation by pulse oximetry (SatTrak, SensorMedics). We measured CPET parameters and lung function parameters including forced expiratory volume in one-second workload (Watt), maximal ventilation during exercise (VE max), ventilatory equivalents for oxygen (VE/VO_2_), Oxygen pulse (O_2_/HR), ventilatory equivalents for carbon dioxide (VE/VCO_2_).

### DNA extraction from stool samples

All samples were processed by Novogene. DNA extraction, library preparation and sequencing was done as described previously [19]. Stool samples were thoroughly mixed with 900 μL of CTAB lysis buffer. All samples were incubated at 65°C for 60 min before being centrifuged at 12000×g for 5 min at 4°C. Supernatants were transferred to fresh 2 mL microcentrifuge tubes and 900 μL of phenol:chloroform:isoamyl alcohol (25:24:1, pH = 6.7; Sigma-Aldrich) was added for each extraction. Samples were mixed thoroughly prior to being incubated at room temperature for 10 min. Phase separation occurred by centrifugation at 12,000×g for 15 min at 4°C, and the upper aqueous phase was re-extracted with a further 900 μL of phenol:chloroform:isoamyl alcohol. Next, samples were centrifuged at 12,000×g for 10 min at 4°C, and the upper aqueous phases were transferred to fresh 2 mL microcentrifuge tubes. The final extraction was performed with 900 μL of chloroform:isoamyl alcohol (24:1), and layer separation occurred by centrifugation at 12,000×g for 15 min at 4°C. Precipitation of DNA was achieved by adding the upper phase from the last extraction step to 450 μL of isopropanol (Sigma-Aldrich) containing 50 μL of 7.5M ammonium acetate (Fisher). Samples were incubated at– 20°C overnight. Samples were centrifuged at 7500×g for 10 min at 4°C, and supernatants were discarded. Finally, DNA pellets were washed three times in 1 mL of 70% (v/v) ethanol (Fisher). The final pellet was air-dried and re-suspended in 200 μL of 75mM TE buffer (pH = 8.0; Sigma-Aldrich).

### Library preparation and sequencing

Sequencing library was generated based on Illumina technologies and followed manufacturer’s recommendations. Index codes were added to each sample. Briefly, the genomic DNA was randomly fragmented to a size of 350 bp, then DNA fragments were narrowly size selected with sample purification beads. The selected fragments were then end polished, A-tailed, and ligated with adapter. These fragments were filtered with beads again and amplified by PCR reaction. At last, the library was analysed for size distribution and quantified using real-time PCR. The library was then to be sequenced on an Illumina platform Novaseq 6000 (Novogene) with paired-end reads of 150 bp.

The concentration of genomic DNA for ITS2 sequencing was determined by Qubit and the DNA quality was checked on the gel. 200 ng of DNA was used as input for PCR reaction with corresponding primer set specifically binding to different hypervariable regions. Each primer set had a unique barcode. PCR product was then run on the gel and DNA fragment with the proper amplification size was cut and purified. The purified PCR product was then used as template for library preparation. The PCR products were pooled together with equal amount and then end polished, A-tailed, and ligated with the adapter. These fragments were filtered with beads again. After PCR reaction, the library was analysed for size distribution and quantified using real-time PCR. The library was then to be sequenced on Hiseq2500.

### Quality control

Quality control of raw reads was performed using the Sunbeam 2.1 pipeline [20]. First, cutadapt [21] version 2.8 was used to remove universal adapter sequences. Next, trimmomatic [22] version 0.36 was used to perform Illumina-specific adapter trimming, window quality trimming (Q5 over 25 nt), and 3’ and 5’ clipping (Q<6). Resulting reads shorter than 36nt were removed. Decontamination of human reads was performed by mapping quality-controlled reads with BWA [23] version 0.7.17 against a masked human reference genome (GRCh38-89). Masking of low entropy regions was performed using BBmask. Reads with 99% coverage and >97% identity to the human reference were removed.

### Taxonomic and functional annotation

Taxonomic annotation was performed using MetaPhlAn2 [24] version 2.7.7 with default settings, generating taxonomic relative abundances.

The PIPITS pipeline [25] version 2.4 was used for taxonomy annotation of fungal Internal Transcribed Spacer (ITS) with default parameters including quality filtering, read-pair merging, ITS2 extraction and chimera removal. Remaining reads were binned based on 97% similarity as operational taxonomic units and aligned to the UNITE fungi database using Mothur classifier [26].

Functional annotation was performed using HUMAnN2 [27] version 0.11.2. In the pipeline, the reads were mapped to the MetaCyc database for pathway annotation, and the UniRef90 database to estimate gene family abundances. These abundances were aggregated into MetaCyc pathway abundances using MinPath based on the MetaCyc database.

### Microbial community diversity analysis

Alpha diversity indices detailing microbial community composition within samples were calculated using the R packages vegan [28] and fossil [29]. For estimating beta diversity reflecting community dissimilarities, Bray-Curtis distances were calculated using R package vegan [28].

### Co-abundance network

For bacterial co-abundance network reconstruction, the species relative abundance table was split into before and after surgical resection samples, and they were processed independently with SparCC [30].

### Correlation analyses with lung function parameters

Partial Spearman’s correlations adjusted for Chronic obstructive pulmonary disease (COPD) and cancer type were determined between significantly different bacterial features (species and pathways), fungal species abundances, and lung function parameters. For visualization only bacterial features with significant correlations (*P*<0.05) and fungal species with significant correlations (*P*<0.05) and absolute correlation coefficient > 0.65 were selected.

### Prediction of VO_2_, recurrence and overall survival (OS)

Prediction of VO_2_, recurrence and OS was performed by calculating microbial balances [31]. A working implementation is available https://github.com/UVic-omics/selbal. A microbial balance is a special kind of log-contrast. Briefly, let X = (X_1_,X_2_,…,X_k_) be a composition with k components. For two disjoint subsets of k_+_ and k_-_ parts, respectively, the balance B of the component sets X_+_ and X_-_ is determined by the formula:

$$B\left(X_{+},X_{-}\right)\propto \frac{1}{k_{+}}\sum_{i\in I_{+}}\text{log}X_{i}-\frac{1}{k_{-}}\sum_{j\in I_{-}}\text{log}X_{j}$$

Feature selection for X_+_ and X_-_ was performed using the function "selbal.cv", which implements an iterative cross-validation procedure to (i) identify the optimal number of components (C_opt_) to be included in the balance and (ii) estimate the importance of the selected components. Only features (bacterial species and MetaCyc pathways) that changed significantly after surgery were used for feature selection.

Since “selbal.cv” is a forward selection process where components are included sequentially at every step, we have a sequence of balances, ranging from C = 2 to C = 20 components. Once C_opt_ has been determined, we apply “selbal.cv” to the whole data set, with the number of taxa C_opt_, and obtain the global balance.

In order to estimate the robustness of the global balance and the importance of selected components, all balances with C_opt_ components obtained in the cross-validation process are retrieved and compared to the global balance, obtaining the relative frequencies of the different balances and the proportion of times that each component was included in a balance. The more often a component was selected, the higher its importance.

### Statistical analysis

Testing for significant differences in alpha diversity between before and after surgical resection was performed using Wilcoxon signed-rank test. To test for significant differences in the microbial composition between before and after surgical resection, beta-diversity was analysed using permutational multivariate analysis of variance (PERMANOVA) as implemented in the function adonis from R package vegan [28].

Bacterial species and MetaCyc pathways were filtered by 10% prevalence across all samples and their relative abundances were used for statistical comparisons of before vs. after surgery. Differentially abundant features were identified by the Wilcoxon signed-rank test and were considered significantly different if *P*<0.05 after FDR correction using the Benjamini-Hochberg procedure.

Species-species correlation coefficients were estimated as the average of 20 inference iterations and 100 permutations were used for the pseudo P-value calculation. A correlation was considered significant if pseudo P<0.05.

Partial Spearman’s correlations between lung function and CPET parameters with bacterial and fungal features, adjusted for Chronic obstructive pulmonary disease (COPD) and cancer type, were performed using R package ppcor [32] and were considered significant if P<0.05.

## Results

### Community taxonomic and functional diversity before and one year after surgical resection

We analysed data from 15 lung cancer patients who underwent lung resection surgery. Our cohort includes a balanced set of men (n = 7) and women (n = 8), and patients with stage I (n = 4), stage II (n = 8) and stage IIIA (n = 3) disease. Tumours recurred in six patients. Detailed patients’ characteristics are shown in S1 Table.

These subjects had an overall good performance status (Eastern Cooperative Group Performance Status 0) at diagnosis.

**Fig 1:**
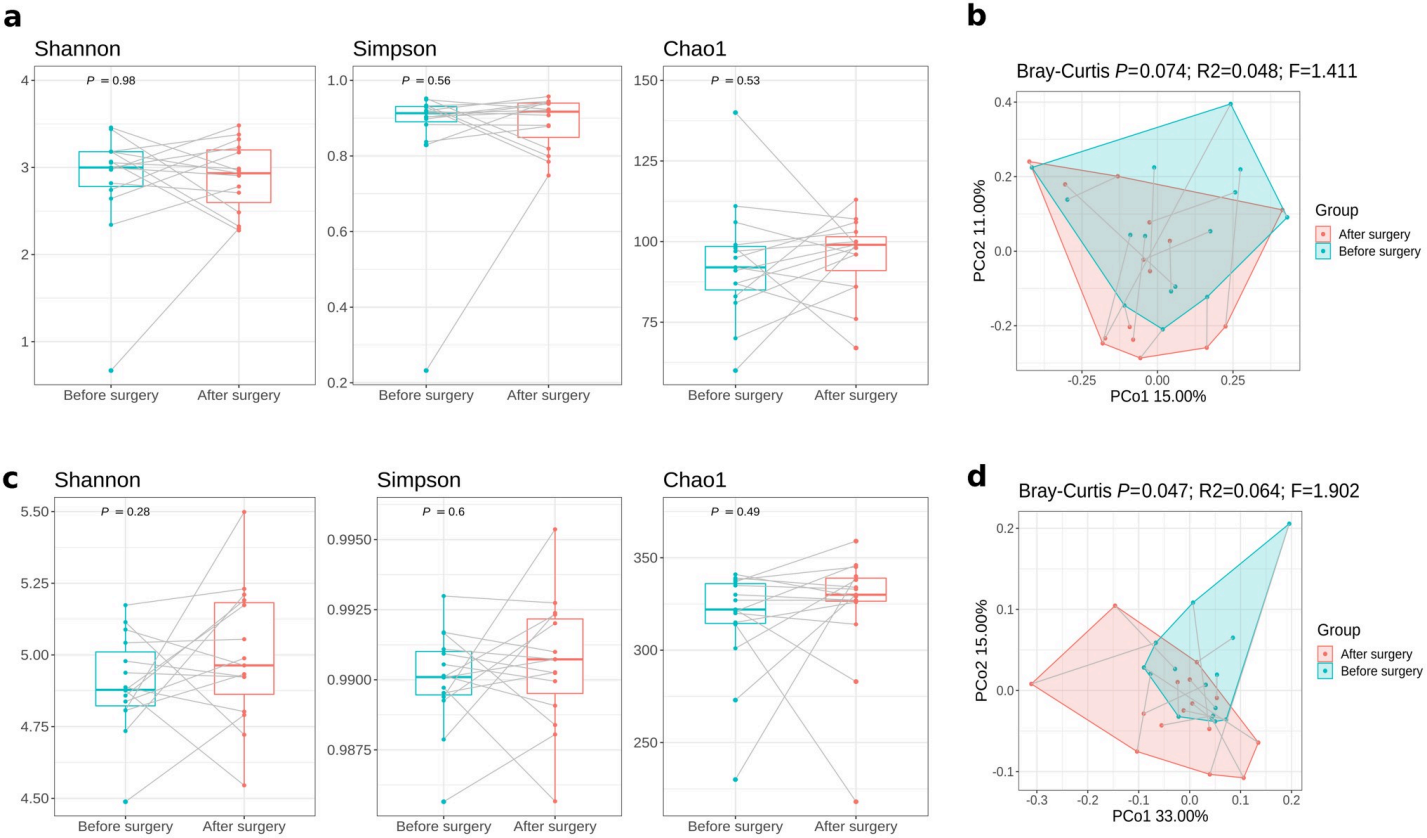
Comparison of the gut microbiota composition before and after surgical resection. (A, C) Boxplots, with median (centrelines), first and third quartiles (box limits) and 1.5x interquartile range (whiskers), showing alpha diversity Shannon, Simpson, and Chao1 indices at (A) bacterial species and (C) bacterial MetaCyc pathways. Gray lines connect samples of the same patient from before and after surgical resection. (B, D) Principal Coordinate Analysis plot based on Bray-Curtis distances before and after surgical resection at (B) bacterial species and (D) bacterial MetaCyc pathways. Gray lines connect the measurement of a patient before and after surgical resection.

The structure and function of the gut microbiome were assessed using shotgun metagenomic sequencing. In total, 337 bacterial species were detected. First, we compared the overall composition of the gut microbiome communities pre- versus post-surgical resection. There were no significant changes in bacterial species alpha diversity over time (Shannon *P* = 0.98, Simpson *P* = 0.56, Chao1 *P* = 0.53; Wilcoxon signed-rank test) (Fig 1A). Bacterial species beta diversity did not show significant differences either, but indicated a trend (Bray-Curtis dissimilarity, *P* = 0.074, R^2^ = 4.8%, PERMANOVA) (Fig 1B). We further compared the species’ beta diversity taking into account the different stages of disease and chemotherapy treatment and found no significant differences (S2A and S2B Fig). Alpha and beta diversity comparisons of recurrent and non-recurrent patients showed no significant differences either, but indicated a trend with non-recurrent patients having higher alpha diversity at both, pre- and post-surgical resection (S2C and S2D Fig).

We used shotgun metagenomic sequencing to further examine the variation of gut bacterial functions. In total, 498 MetaCyc pathways were retrieved. We did not observe significant differences in MetaCyc alpha diversity evenness and richness after surgical resection (Shannon *P* = 0.28, Simpson *P* = 0.6, Chao1 *P* = 0.49; Wilcoxon signed-rank test) (Fig 1C). However, we found a significant difference in bacterial functional profiles (Bray-Curtis dissimilarity) from post-surgical samples compared with pre-resection samples (*P* = 0.047, R^2^ = 6.4%, PERMANOVA) (Fig 1D).

### Alterations of gut microbial species, co-abundance network and functions one year after surgical resection

**Fig 2:**
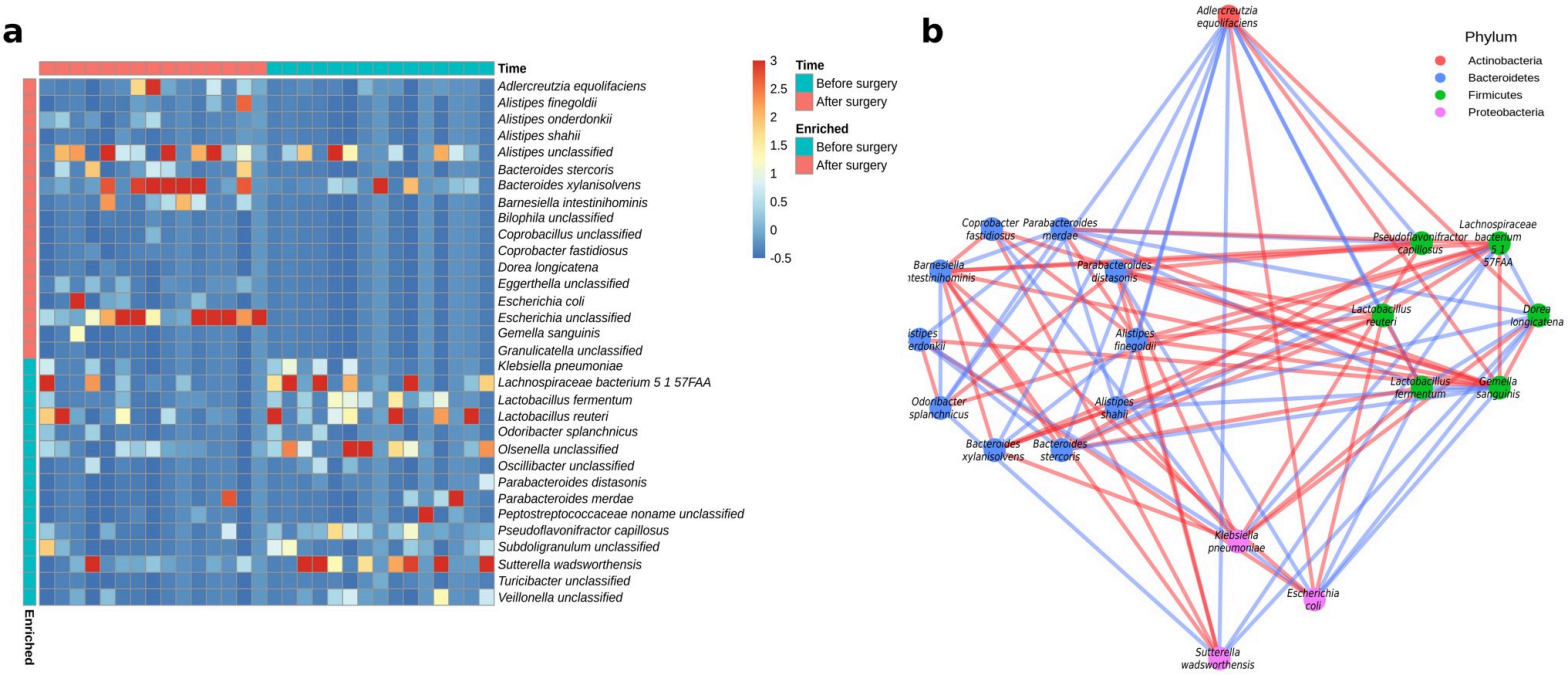
Taxonomic analysis of the gut microbiome in response to surgical resection. (A) Heatmap of differentially abundant bacterial species (*P*<0.05, Wilcoxon signed-rank test) before and after surgical resection. Red and blue in the far-left column indicates increased and decreased relative abundance, respectively. (B) Co-abundance network of bacterial species using SparCC [30]. Only correlations between differentially abundant bacterial species (*P*<0.05, Wilcoxon signed-rank test) that changed direction were used for network construction. The nodes are coloured based on their affiliated phyla. Edge colour indicates either correlations that changed from positive to negative (blue) or from negative to positive (red).

Next, we focused on taxonomic changes at the species level. We investigated which prevalent species changed significantly in relative abundance post-surgical resection. Thirty-two bacterial species showed significant changes after surgical resection compared to pre-surgical resection (*P*<0.05, Wilcoxon signed-rank test) (S2 Table). Of these, 15 were enriched pre-surgical resection, and 17 were enriched post-surgical resection (Fig 2A). We observed a significant decrease in the abundance of atypical opportunistic pathogens, such as *Klebsiella pneumoniae* [33], and *Odoribacter splanchnicus* [34]. These species are usually harmless within the gut of their host, but cause infection outside this niche. Other opportunistic pathogens like *Sutterella wadsworthensis* [35] decreased in abundance. Several species from *Alistipes* and *Bacteroides* genus, related to a healthy microbiome, increased significantly in abundance. These species have been associated with the production of SCFAs [36, 37].

We estimated relationships among gut microbes by constructing co-abundance networks based on bacterial species relative abundance (using SparCC [30]) for pre- and post-surgical resection groups, respectively. For evaluating the impact of surgery on the microbial community structure, we focused only on correlations between significant species that changed either from positive to negative or vice versa (Fig 2B). Overall, co-occurrence relationships were observed within and between phylum Actinobacteria, Bacteroidetes, Firmicutes, and Proteobacteria. We found more correlations changing from negative to positive post-surgical resection (47) than from positive to negative (37). The species that showed the most changing correlations were *Gemella sanguinis*, *Adlercreutzia equolifaciens*, *Lachnospiraceae bacterium 5 1 57FAA*, *Parabacteroides merdae*, *K*. *pneumoniae* and *Barnesiella intestinihominis*.

**Fig 3:**
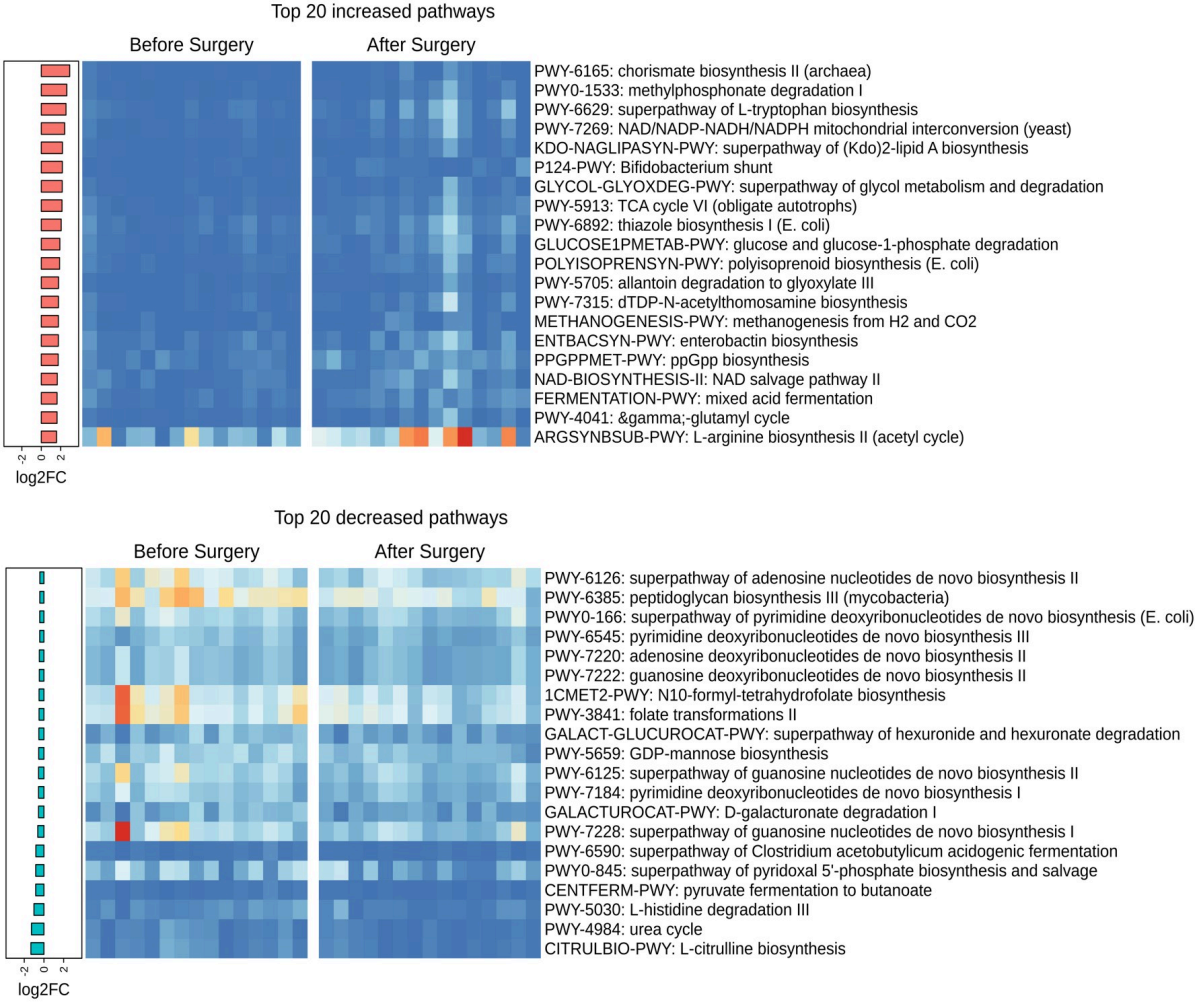
Functional analysis of the gut microbiome in response to surgical resection. Heatmaps of the top 20 increased (top) and decreased (bottom) differentially abundant bacterial MetaCyc pathways (*P*<0.05, Wilcoxon signed-rank test). Histogram (left panel) shows the pathways ranked by variation (log2 fold-change after surgical resection).

Furthermore, 78 MetaCyc pathways differed significantly post-surgical resection compared to pre-surgical resection (*P*<0.05, Wilcoxon signed-rank test) (S3 Table) with 22 pathways enriched pre-surgical resection and 56 pathways post-surgical resection. The pathways with the highest increase were mostly related to the generation of precursor metabolites and energy and the biosynthesis of cofactors, electron carriers, and vitamins, whereas the pathways with the highest decrease were mostly related to core bacterial functions such as nucleoside and nucleotide biosynthesis (Fig 3).

### Associations of bacteria and their function with lung function and CPET parameters

**Fig 4:**
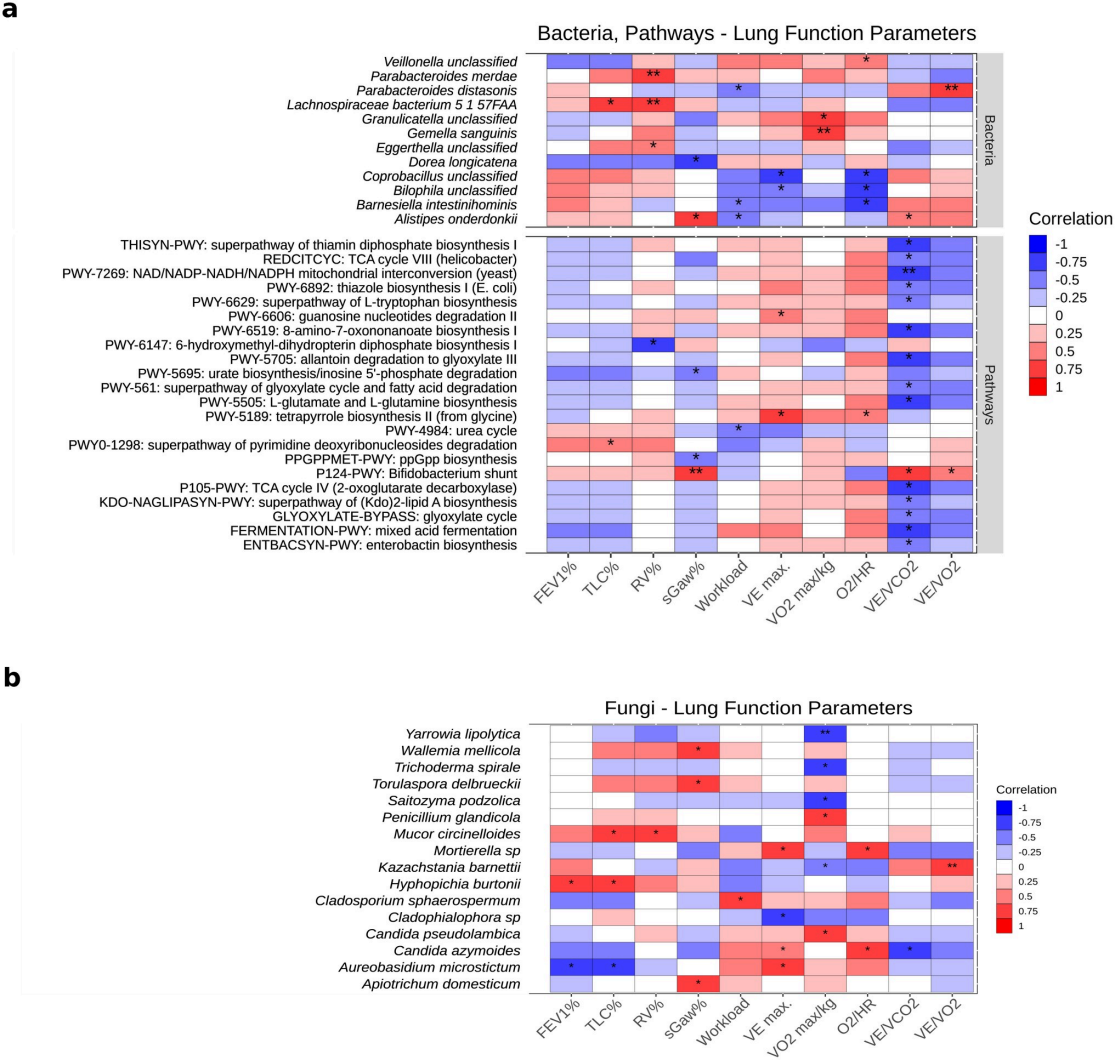
Correlations between taxonomic and functional profiles and CPET. (A) Heatmap of partial Spearman’s rank correlation analysis between bacterial species and bacterial MetaCyc pathways versus lung function parameters adjusting for COPD and cancer type. Only differentially abundant species and pathways (*P*<0.05, Wilcoxon signed-rank test) were used. (B) Heatmap of partial Spearman’s rank correlation analysis between fungal species versus lung function parameters adjusting for COPD and cancer type. (A, B) Cell colour indicates either negative correlation (blue) or positive correlation (red). Only species and pathways with significant correlations ((A) *P*<0.05; (B) *P*<0.05, absolute correlation coefficient > 0.65) are shown (*P<0.05, **P<0.01, ***P<0.001).

One year post-surgery, we investigated the significant associations between differentially abundant taxonomic and functional features with lung function and CPET parameters (FEV1%, TLC%, RV%, workload (Watt), VE, VO_2_, O_2_/HR, VE/VCO_2_, VE/VO_2_) adjusting for cofounders such as COPD and cancer type. In total, 12 bacterial species were significantly correlated (*P*<0.05) with lung function and CPET parameters. From these species, six were positively correlated with parameters: *Veillonella unclassified* (O_2_/HR), *P*. *merdae* (RV%), *L*. *bacterium 5 1 57FAA* (TLC% and RV%), *Granulicatella unclassified* (VO_2_), *G*. *sanguinis* (VO_2_) and *Eggerthella unclassified* (RV%), whereas four species showed negative correlations: *Dorea longicatena* (Specific Airway Conductance (sGaw%)), *Coprobacillus unclassified* (VE and O_2_/HR), *Bilophila unclassified* (VE and O_2_/HR), *and B*. *intestinihominis* (workload and O_2_/HR). *Parabacteroides distasonis* was positively correlated with VE/VO_2_ and negatively correlated with workload while *Alistipes onderdonkii* was positively correlated with sGaw% and VE/VCO_2_ and negatively correlated with workload (Fig 4A). We also investigated the relations between post-surgery changes in the abundance of species with post-surgery changes in lung function parameters (FEV, FEV1% and FEV/FVC). We identified 10 bacterial species, including species correlated with CPET parameters, such as *P*. *distasonis* and *B*. *intestinihominis* significantly correlated with changes in lung function parameters (S3A Fig).

Of the significantly differentially abundant MetaCyc pathways, four were positively associated with at least one of TLC%, sGaw%, VE, O_2_/HR, VE/VCO_2_, and VE/VO_2_, including pathways for generation of precursor metabolites and energy, nucleoside and nucleotide degradation, and tetrapyrrole biosynthesis. Eighteen significantly differentially abundant MetaCyc pathways showed a negative correlation with lung function parameters (RV%, sGaw%, workload, and VE/VCO_2_), including pathways responsible for compound degradation, utilization, and assimilation (N = 3), cofactor, electron carrier, and vitamin biosynthesis (N = 4), amino acid biosynthesis (N = 2), compound biosynthesis (N = 4), and generation of precursor metabolites and energy (N = 5) (Fig 4A) (S4 Table). Post-surgery changes in the abundance of 4 pathways, related to amino acid biosynthesis, cofactor, electron carrier, and vitamin biosynthesis, and compound degradation, utilization and assimilation, were positively associated with post-surgery changes in lung function parameters (S3A Fig). In contrast, post-surgery changes in the abundance of two pathways were negatively associated with post-surgery changes in lung function parameters (S3A Fig).

We compared the abundance of aerobic and anaerobic species before and after surgery and found a strong, significant decrease in the abundance of anaerobes after surgical resection (r = 68.9%, P = 0.0054, Wilcoxon signed-rank test) (S4A Fig). When stratifying for recurrent and non-recurrent patients separately, recurrent patients showed a very strong, significant decrease in the abundance of anaerobes (r = 89.9%, P = 0.031, Wilcoxon signed-rank test) (S4B Fig). Non-recurrent showed only a trend (r = 54.5%, P = 0.15, Wilcoxon signed-rank test). Given the difference in effect, these findings imply a potential role of anaerobe bacteria in tumour suppression.

At last, we tried to predict VO_2_, tumour recurrence, and overall survival (OS) from either (a) bacterial or (b) MetaCyc pathway relative abundances (S5 Fig, S5 Table). We only looked at features with significant change post-surgical resection. We selected features whose microbial balance [31] has a high linear correlation to target response. For VO_2_, the balance of only six bacterial species positively correlates with a Pearson correlation coefficient of *r* = 0.78 (*P* = 6E-4). Here, the balance was termined to be the ratio of *A*. *equolifaciens* and *Alistipes* abundances (both increased significantly after surgery) to *P*. *distasonis*, *P*. *merdae* (both decreased significantly after surgery) and *G*. *sanguinis* abundances. The balance of 7 MetaCyc pathways correlates even better with *r* = 0.85 (P = 7E-5). Functions for carbohydrate utilization, fatty acid oxidation and arginine production were selected. Interesting, an increase in VO_2_ coincides with an increase in the pathway "GABA shunt", in which 4-aminobutyrate (GABA) is produced from glutamate and further metabolized to succinate. Improvements in exercise tolerance and treatment outcome were associated with butyrate producing species. Succinate is involved in several metabolic processes. Importantly, it is involved in the control of reactive oxygen species and tumorigenesis [38]. In contrast, functions for metabolizing glucose and fucose may have a negative impact on VO_2_. In contrast to previous findings [8], the F/B ratio was not correlated or predictive of VO_2_. However, the ratio of Proteobacteria against Euryarchaeota and Actinobacteria correlated with *r* = 0.56 (p = 0.032), implying a potential link between members of these phyla and VO_2_.

For prediction of tumour recurrence vs non-recurrence, patients can be accurately classified by a balance of three bacteria (CV-AUC = 95%, Acc: 100%). *O*. *splanchnicus* is hereby associated with tumour free outcome, whereas *G*. *sanguinis* and *Olsenella* were associated with tumour recurrence. A balance of two MetaCyc functions was also predictive of recurrence (CV-AUC: 90%, Acc: 100%). Hereby, L-alanine biosynthesis was associated with positive outcome. Arginine and polyamine biosynthesis were predicted to increase tumour recurrence.

For OS, a balance of 5 MetaCyc pathways correlated well (r = 87%, *P* = 2e-5). Interestingly, guanosine nucleotide de novo synthesis pathways were associated with improved OS, while adenosine and pyrimidine nucleotide de novo synthesis to decreased OS.

### Associations of fungal species and lung function and CPET parameters

Besides the bacterial composition, we also attempted to associate the mycobiome with the lung function parameters. We built high-quality libraries for ITS2 sequencing for samples collected post-surgical resection (N = 15) and estimated the relative fungal abundance using the PIPITS pipeline [25]. In total, we identified 124 genera and 189 species. From these, 16 fungal species showed strong significant correlations (*P*<0.05, absolute correlation coefficient > 0.65) with lung function and CPET parameters (FEV1%, TLC%, sGaw%, Workload, VE, VO_2_, and O_2_/HR) (Fig 4B). *Penicillium glandicola* and *Candida pseudolambica* were positively correlated with VO_2_, whereas *Yarrowia lipolytica*, *Trichoderma spirale*, and *Saitozyma podzolica* showed negative correlations (S5 Table).

## Discussion

CPET is a non-invasive method to test the overall condition of lung cancer patients who underwent lung resection surgery. CPET provides a comprehensive assessment of the exercise response and reflects the metabolic interactions of different organ systems. Shotgun metagenomic sequencing allows taxonomic and functional annotation of the microbiome, thus it is the most comprehensive method for microbiome characterization.

Early metabolic changes might predict postoperative physical condition and outcome earlier than radiographic changes. The gut-lung axis plays a critical role in metabolic functional changes, and therefore, we hypothesized that the gut microbiome might be associated with different workloads and other CPET parameters.

Previous studies have shown that colorectal cancer patients have a disturbed gut microbial composition with a low abundance of species producing butyrate, such as species from the *Roseburia* and *Lachnospiraceae* genera, which may alter gene expression in healthy and cancerous cells [39]. In colorectal cancer cells, a dysfunction of the mitochondria contributes to an accumulation of butyrate in the cytosol and inhibition of histone deacetylases, resulting in the downregulation of proliferation and apoptosis pathways [39]. These changes may contribute to a decrease in tumour size and the probability of metastasis.

In our study, we observed post-surgical increases in (a) SCFA producing bacterial species and (b) pathways involved in carbohydrate, alcohol metabolism, and vitamin B production. These findings suggest a possible relationship between bacterial communities capable of metabolizing carbohydrates and alcohols into chemical compounds. The compounds involved in these pathways are considered important (a) to improve cell membrane barrier, (b) to act as precursor metabolites for human hormones such as serotonin and melatonin, and (c) for vitamin B_1_ and B_3_ production. Furthermore, we predicted a negative association between *O*. *splanchnicus* and tumor recurrence. *O*. *splanchnicus* is a potent butyrate producing species [40] by fermentation of L-Lysing (BioCyc P163-PWY [41]) and integral part of a healthy gut microbiome [42]. To improve CPET VO_2_ and supress tumour recurrence, enriching the host microbiome with butyrate producing species and decreasing monosaccharide metabolizing species may improve treatment outcome.

From our study, we conclude that *A*. *equolifaciens*, *Alistipes*, *L*. *bacterium 5 1 57FAA* and *P*. *merdae* may be beneficial species for improved recovery of overall physical condition and lung capacity. These species were positively correlated with several lung function parameters or predictive of VO_2_. In contrast, *G*. *sanguinis* was associated with decreased VO_2_ and increased tumor recurrence. *D*. *longicatena* was associated with a decrease in lung function parameters, suggesting a negative impact of these two bacterial species on the recovery of lung capacity. Both *L*. *bacterium 5 1 57FAA* and *P*. *merdae* were negatively correlated with *D*. *longicatena*, implying that these two species may compete with *D*. *longicatena* for gut colonization and could therefore improve health post-surgery. While *O*. *splanchnicus* was not directly associated to improved lung function, a potential effect on tumour suppression should be worth further investigation. We observed a significant decrease in relative abundance of this bacterial species after resection. Like other SCFA producers, high fibber diets are required for the fermentation, proliferation and hence SCFA production. In the future, it should be worth to investigate if SCFA and other metabolites produced by *O*. *splanchnicus* are taken up by their human host and how these may influence tumour tissue.

Our study’s limitation includes that in the real-life setting even though initial case numbers of recruited patients were high, the final numbers of patients with overall good clinical condition and available samples were low. To our best knowledge, the sample size is comparable to other studies of comparably unique conditions. Accordingly, this study did not allow us to draw robust conclusions on outcome-related biomarkers; however, it enabled us to meet our primary aim to study metabolic interactions. Another limitation is that the causality of metabolic and microbiome changes is not clear and follow-up studies with larger cohorts are needed.

We conclude that there are bacterial metabolic pathways that might be associated to increased oxygen uptake and exercise tolerance in patients one year after lung resection surgery. To our knowledge, this is the first study on microbiome functionality correlations with CPET, a highly specialized stress test with parameters that provide a unique detailed opportunity to study not only taxa but also metabolic interactions. Also, restoring specific bacteria might provide future therapeutic targets. Additionally, our findings highlight the potential of examining dynamic function parameters compared with traditional static metrics such as basic lung function and radiographic assessment in order to assess different organ systems’ metabolic interactions. In this unique setting, the gut microbiota provided useful information on associations of exercise tolerance. We hypothesize that outcomes after lung resection surgery might be associated with distinct metabolic pathways that need to be confirmed in larger datasets. We offer critical postoperative metabolic and microbiome taxa and functional changes that are associated with distinct patient physical conditions and hopefully provide a reasonable basis for future studies aiming to increase patient outcomes.

Future studies in similar unique datasets are needed to confirm our findings and the modulation of gut microbiota, including butyrate producing taxa to increase long-term benefit from lung resection surgery.

## Supporting information

S1 Fig Study design of surgically resected lung cancer patient cohort. (PDF)

S2 Fig Comparison of the gut microbiota composition pre- and post-surgery. (A, B) Principal Coordinate Analysis plot based on Bray-Curtis distances of (A) the different stages and (B) chemotherapy treatment. (C) Boxplots, with median (centrelines), first and third quartiles (box limits) and 1.5x interquartile range (whiskers), showing alpha diversity Shannon, Simpson, and Chao1 indices of recurrent and non-recurrent patients. (D) Principal Coordinate Analysis plot based on Bray-Curtis distances pre- and post-surgery of recurrent and non-recurrent patients.
(PDF)

S3 Fig Correlations between taxonomic and functional profiles and CPET. (A) Heatmap of partial Spearman’s rank correlation analysis between the fold-change of bacterial species and bacterial MetaCyc pathways versus the fold-change lung function parameters adjusting for COPD and cancer type. Only differentially abundant species and pathways (P<0.05, Wilcoxon signed-rank test) were used. (B) Heatmap of partial Spearman’s rank correlation analysis between fungal species versus the fold-change of lung function parameters adjusting for COPD and cancer type. (A-B) Cell color indicates either negative correlation (blue) or positive correlation (red). Only species and pathways with significant correlations (P<0.05) are shown (*P<0.05, **P<0.01, ***P<0.001).
(PDF)

S4 Fig Prediction of VO2, tumour recurrence, and overall survival (OS). Prediction of VO_2_, tumour recurrence, and overall survival (OS) from bacterial species (left), bacterial phyla (middle) or MetaCyc pathways (right) relative abundances.
(PDF)

S5 Fig Abundance of aerobic and anaerobic species. (A, B) Boxplots, with median (centrerelines), first and third quartiles (box limits) and 1.5x interquartile range (whiskers), showing the abundance of aerobic and anaerobic species. Gray lines connect samples of the same patient from before and after surgical resection.
(PDF)

S1 Table Major patients’ characteristics. (XLSX)

S2 Table Species Wilcoxon signed-rank test results. (TSV)

S3 Table Pathways Wilcoxon signed-rank test results. (TSV)

S4 Table Partial Spearman’s correlation results of bacterial species and pathways with lung function and CPET parameters. (TSV)

S5 Table VO_2_, recurrence and overall survival predictions. (XLSX)

S6 Table Partial Spearman’s correlation results of fungal species with lung function and CPET parameters. (TSV)
