# Supplementary material for: Gut microbiome functionality might be associated with exercise tolerance and recurrence of resected early-stage lung cancer patients
Source: PLoS One. 2021 Nov 18;16(11):e0259898. doi: 10.1371/journal.pone.0259898 (PMC8601557; doi:10.1371/journal.pone.0259898)
Supplement: S1 Fig — (PDF) [file pone.0259898.s001.pdf]

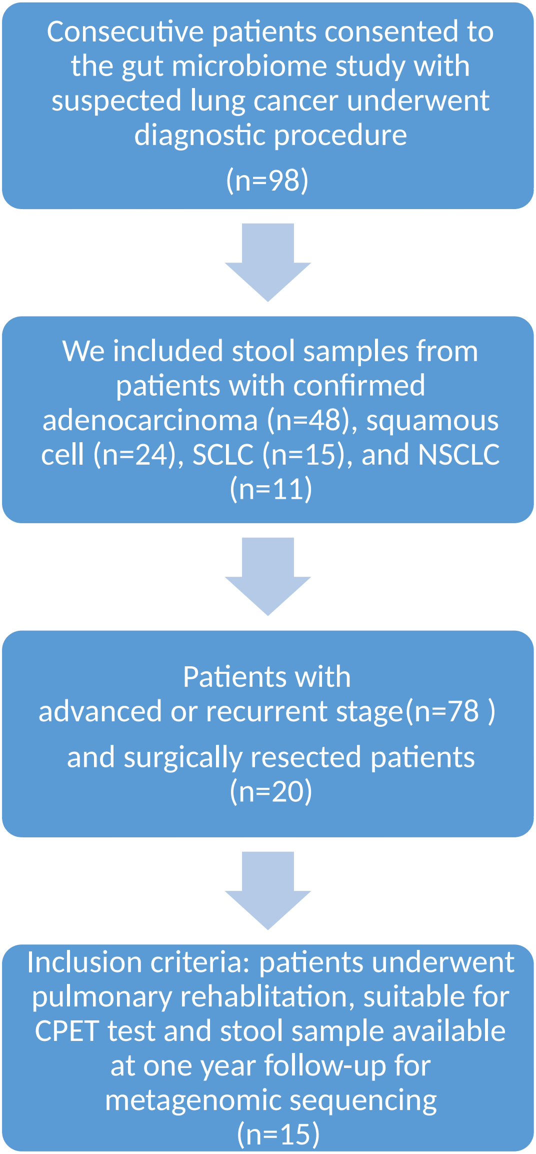
Consecutive patients consented to the gut microbiome study with suspected lung cancer underwent diagnostic procedure
(n=98)
We included stool samples from patients with confirmed adenocarcinoma (n=48), squamous cell (n=24), SCLC (n=15), and NSCLC (n=11)
Patients with advanced or recurrent stage(n=78)
and surgically resected patients (n=20)
Inclusion criteria: patients underwent pulmonary rehablitation, suitable for CPET test and stool sample available at one year follow-up for metagenomic sequencing
(n=15)
