# Supplementary figures and images for: Gut microbiome functionality might be associated with exercise tolerance and recurrence of resected early-stage lung cancer patients
Source: PLoS One. 2021 Nov 18;16(11):e0259898. doi: 10.1371/journal.pone.0259898 (PMC8601557; doi:10.1371/journal.pone.0259898)

**a**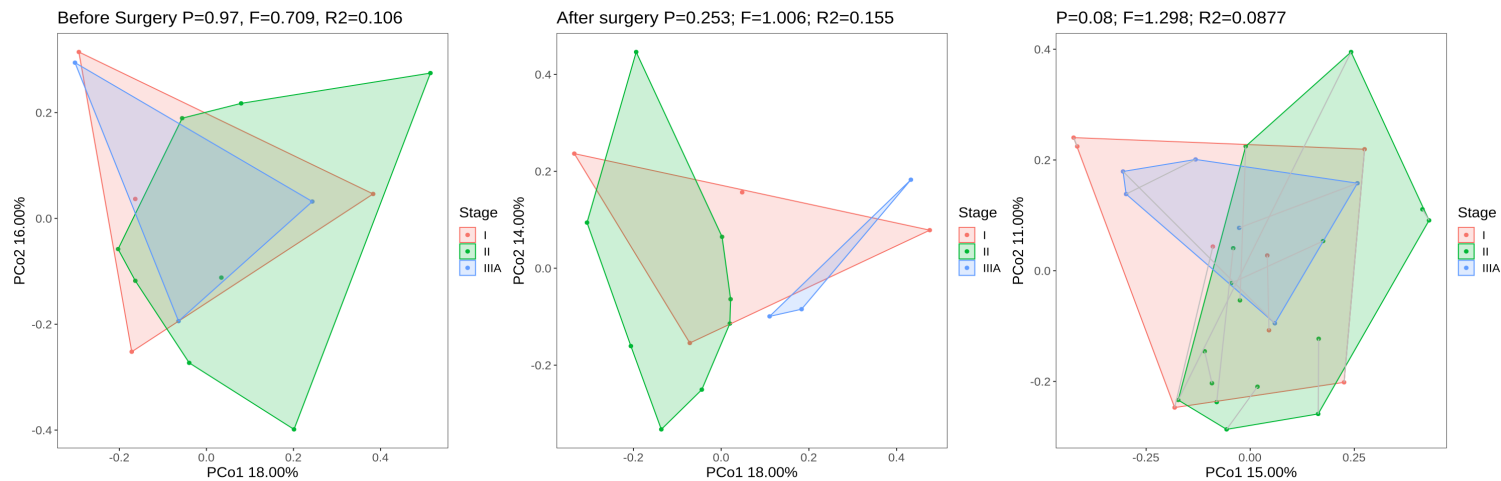**b**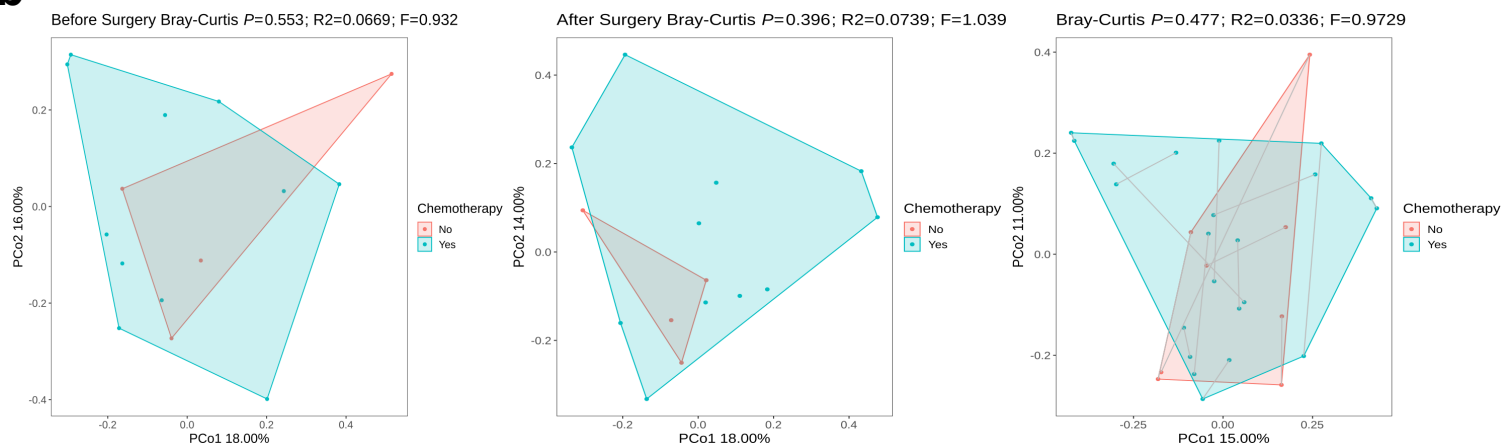**c**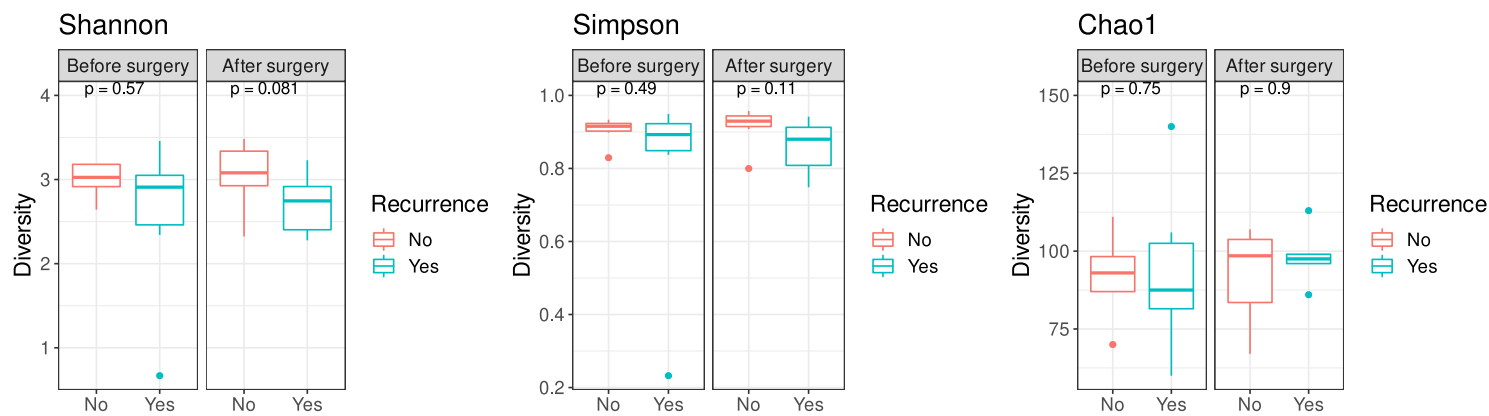**d**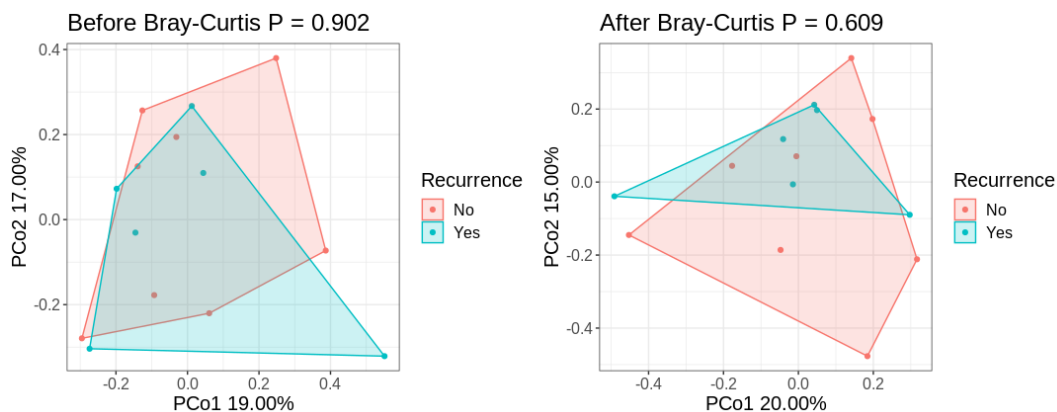

Supplement: S2 Fig — (A, B) Principal Coordinate Analysis plot based on Bray-Curtis distances of (A) the different stages and (B) chemotherapy treatment. (C) Boxplots, with median (centrelines), first and third quartiles (box limits) and 1.5x interquartile range (whiskers), showing alpha diversity Shannon, Simpson, and Chao1 indices of recurrent and non-recurrent patients. (D) Principal Coordinate Analysis plot based on Bray-Curtis distances pre- and post-surgery of recurrent and non-recurrent patients. (PDF) [file pone.0259898.s002.pdf]

## Bacteria, Pathways - Lung Function Parameters

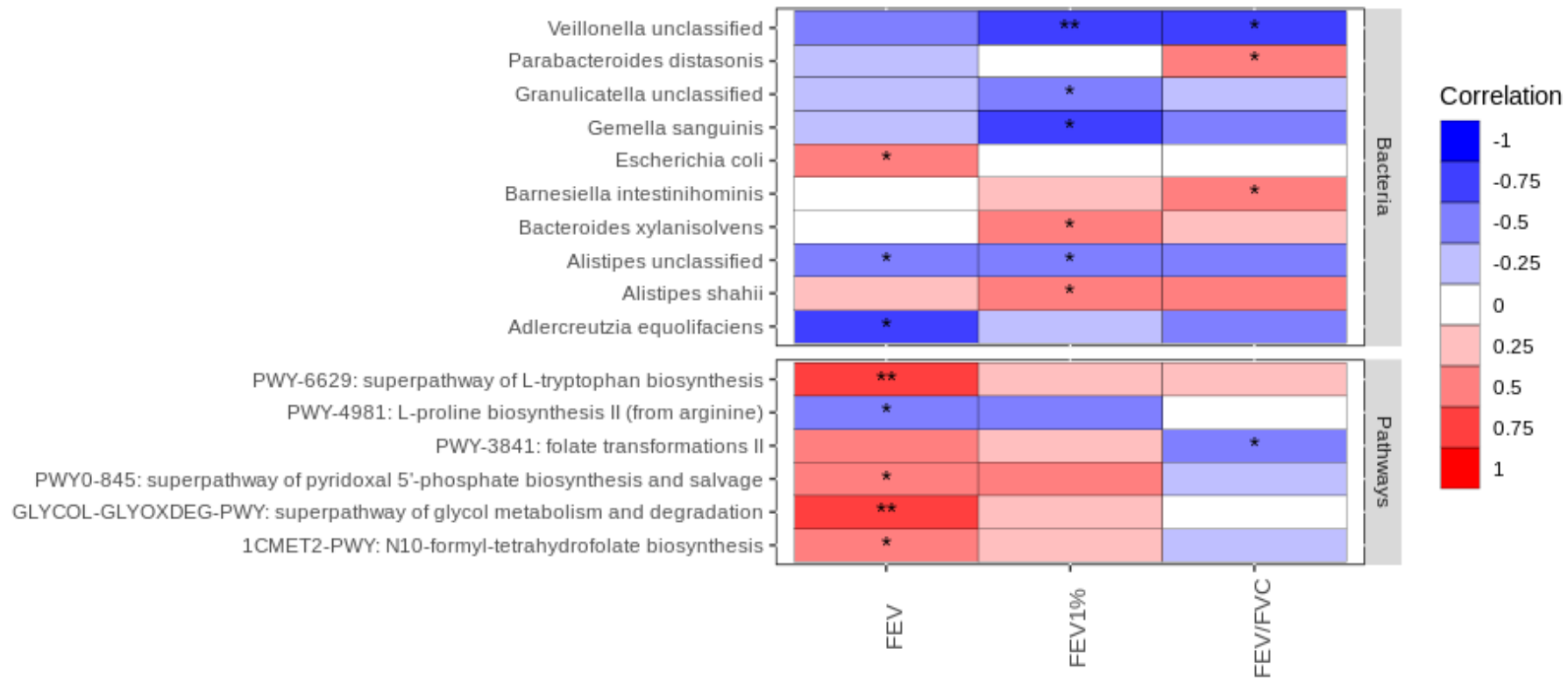

## Fungi - Lung Function Parameters

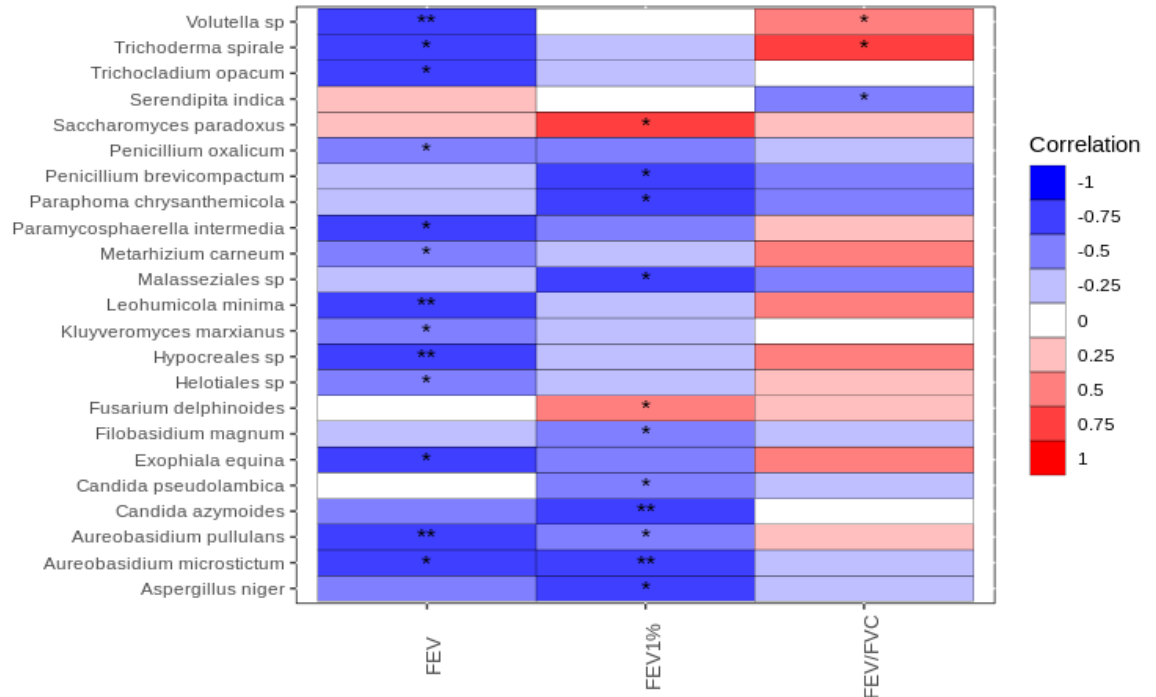

Supplement: S3 Fig — (A) Heatmap of partial Spearman’s rank correlation analysis between the fold-change of bacterial species and bacterial MetaCyc pathways versus the fold-change lung function parameters adjusting for COPD and cancer type. Only differentially abundant species and pathways (P<0.05, Wilcoxon signed-rank test) were used. (B) Heatmap of partial Spearman’s rank correlation analysis between fungal species versus the fold-change of lung function parameters adjusting for COPD and cancer type. (A-B) Cell color indicates either negative correlation (blue) or positive correlation (red). Only species and pathways with significant correlations (P<0.05) are shown (*P<0.05, **P<0.01, ***P<0.001). (PDF) [file pone.0259898.s003.pdf]

**a**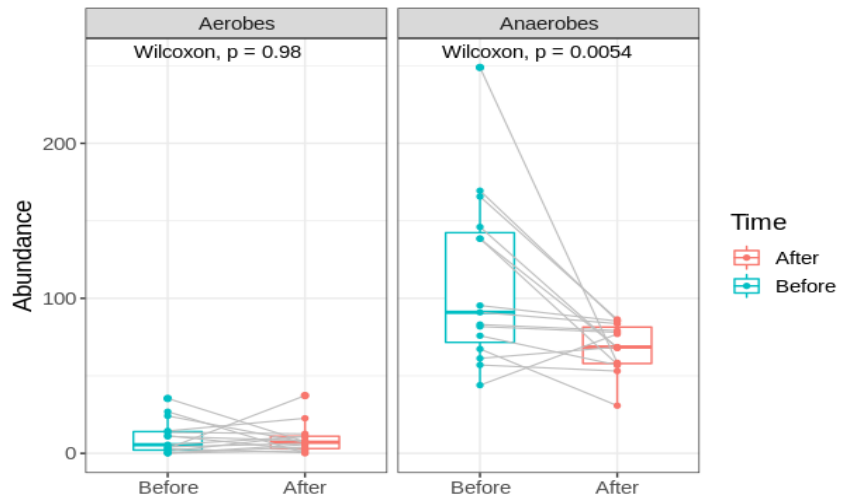**b**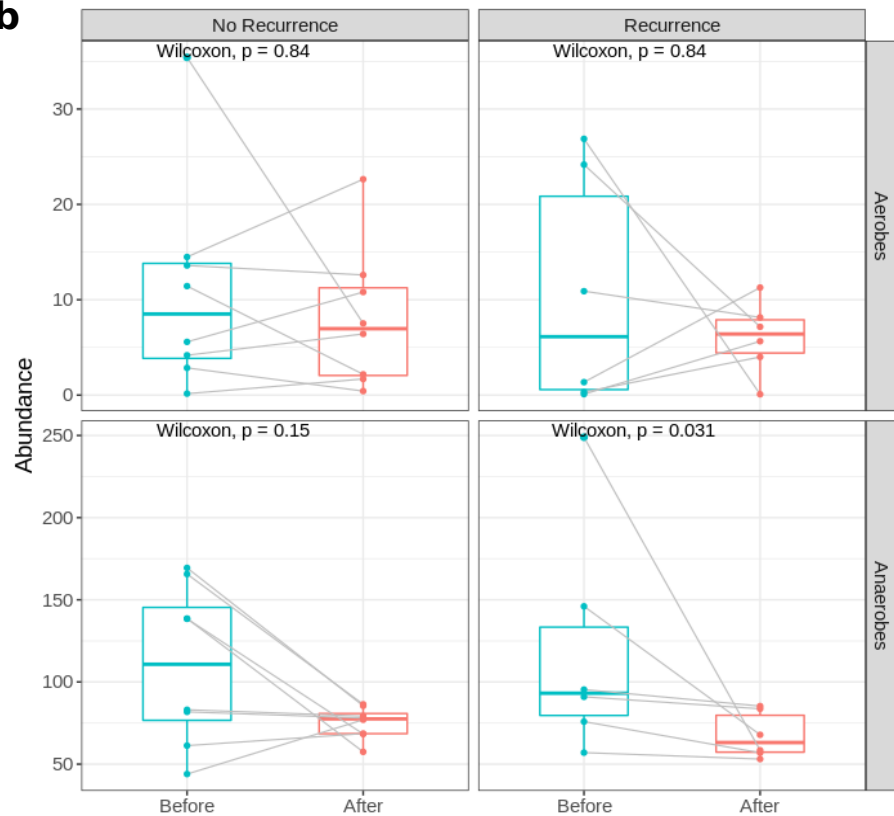

Supplement: S4 Fig — Prediction of VO2, tumour recurrence, and overall survival (OS) from bacterial species (left), bacterial phyla (middle) or MetaCyc pathways (right) relative abundances. (PDF) [file pone.0259898.s004.pdf]

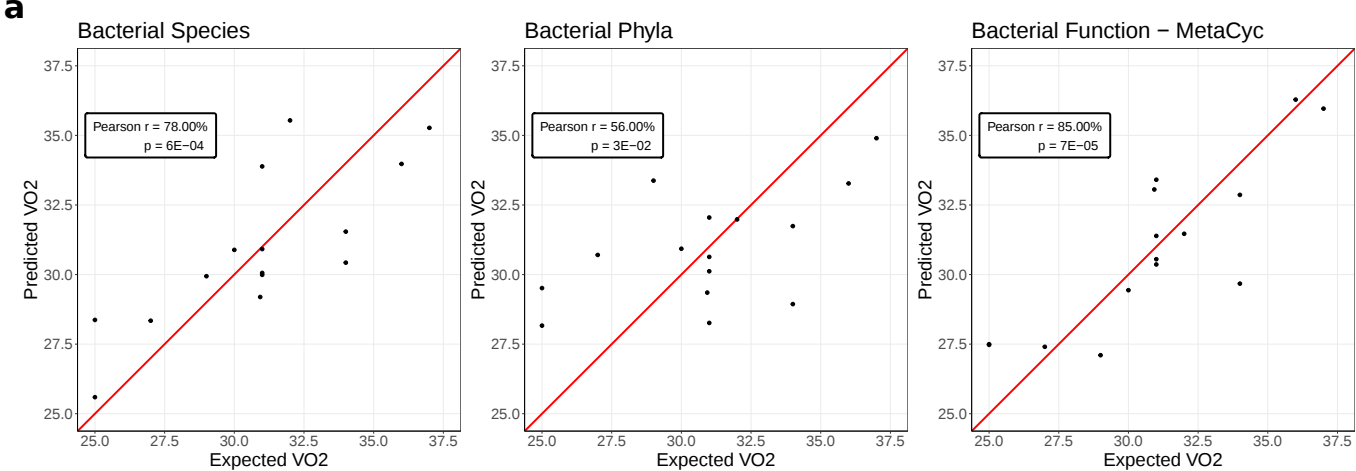

**b**

**Recurrence**

ACC: 100%, CV-AUC: 95%

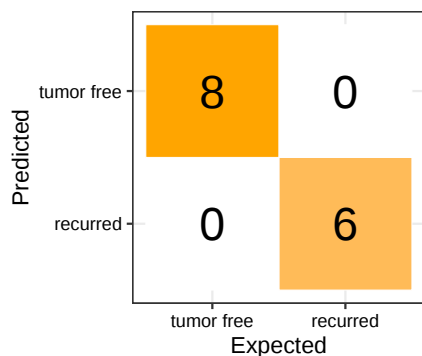

**Recurrence**

ACC: 92.86%, CV-AUC: 85%

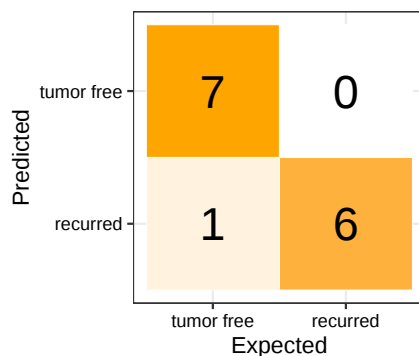

**Recurrence**

ACC: 100%, CV-AUC: 90%

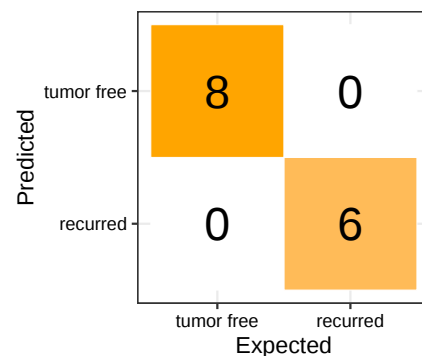

**c**

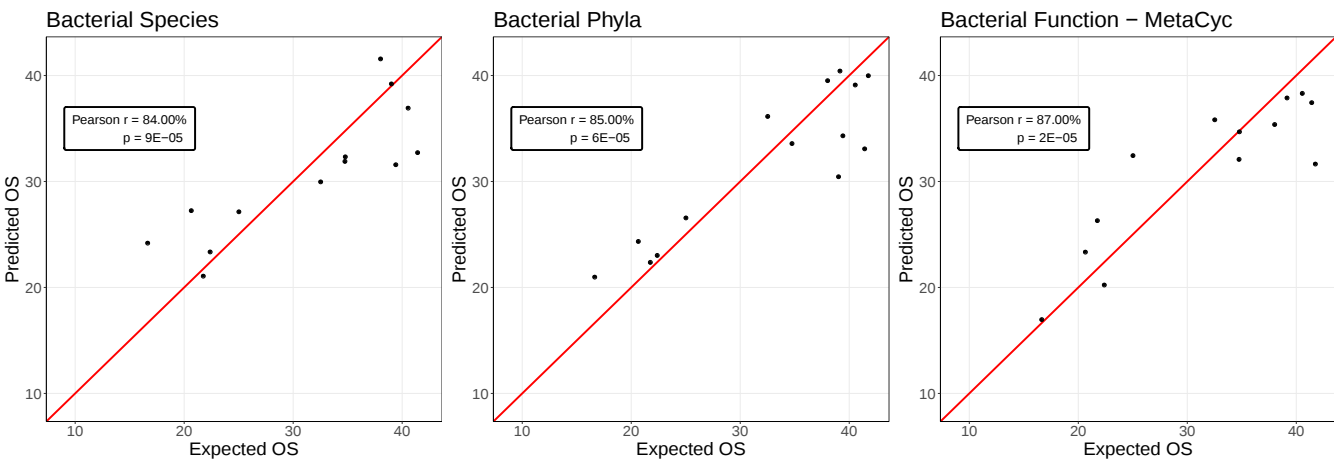

Supplement: S5 Fig — (A, B) Boxplots, with median (centrerelines), first and third quartiles (box limits) and 1.5x interquartile range (whiskers), showing the abundance of aerobic and anaerobic species. Gray lines connect samples of the same patient from before and after surgical resection. (PDF) [file pone.0259898.s005.pdf]
